# Book Notices

**Published:** 1880-07-20

**Authors:** 


### *BOOK NOTICES.*

HOMEOPATHY—WHAT IS IT? A statement and review of its doctrines and practice by A. B. Palmer, M.D., Prof. of Pathology and Practice of Medicine and Sugery in the University of Michigan, etc.: Detroit, Geo. S. Davis, Medical Publisher.

A work of 104 octavo pages, neatly gotten up.

"The work as now presented, says the author, is the result of a careful and candid examination of the essential doctrines of Homeopathy as taught by its founder and authortative expounders, and is a condensed statement of these doctrines. A great many practitioners throughout the country doubtless desire correct information on this subject, and will gladly welcome Dr. Palmer's timely and valuable book.

HEALTH AND HEALTHY HOMES: A Guide to Domestic Hygiene, by Geo. Wilson, M. A. M. D., Medical Officer of Health for Mid-Warwickshire sanitary district and Author of Handbook of Hygiene and Sanitary Science (fourth edition), with notes and additions by J. G. Richardson, M. D., Professor of Hygiene in the University of Pennsylvania, etc., etc. Philadelphia, Pressley Blakiston, 1012 Walnut St., 1880.

This is a neatly gotten up book of 314 pages. The work is instructive and interesting, and treats of a subject too little understood and too much neglected. It will well repay perusal.

PATHOGENETIC OUTLINES OF HOMEOPATHIC DRUGS, by Dr. Med. Carl Hemegke, of Leipzig. Translated from the German by Emil Tietze, M. D., of Philadelphia. Boericke & Tafel, New York, 145 Grant St. Philadelphia, 635 Arch St., 1880.

A work of 375 pages octavo, neatly printed. It is well for the regular physician to be posted upon all subjects. It is due to the Homeopathist to give him a fair hearing and to examine his theory as proclaimed by himself in his standard works. The author in the work before us proposes to give us the "essential outlines of the homeopathic method of cure, in order to meet various erroneous opinions regarding homeopathy, which are promulgated both among physicians and laymen." A repertory is added "presenting to the reader a group of remedies standing in specific relation to certain organs and tissue-systems, and to direct the attention to the comparison of the characteristic features of a definite category of drugs," &c.

A TREATISE ON FOREIGN BODIES IN SURGICAL PRACTICE, by Alfred Poulet, M. D., Ajt. Surg-Maj., Inspector of the School for Medicine at Val-DeGrace. Vol. II., pp. 320. New York, Wm. Wood & Co., 27 Great Jones St.

This is the second volume of a work previously noticed, and is exceedingly useful and practical, treating of foreign bodies in the air passages —the urinary passages, male and female ; in the uterus, in the ear, in the nose, etc.

A HAND-BOOK OF PHYSICAL DIAGNOSIS: Comprising the Throat, Thorax and Abdomen, by Dr. Paul Guttman, Private Docent in Medicine, University of Berlin. Translated from the third German Edition by Alexander Napier, M.D., Fel. Fac. Phys. and Surg., Gasgow: with a Colored Plate and Eighty-nine Fine Wood Engravings. New York, Wm. Wood & Co., 27 Great Jones St., 1880.

A valuable, interesting and instructive work of 335 octavo pages, upon a department little understood by the large majority of general practitioners, as it "presents a concise description of the various methods pursued in the chemical examinations of the thoracic and abdominal organs in health and disease, and an estimate of the diagnostic value of the results so obtained. The examination of the larynx is treated of in an apendix."

ELEMENTARY PRINCIPLES OF SCIENTIFIC AGRICULTURE, by N. L. Lupton, L.L.D., Prof. of Chemistry in Vanderbilt University, Nashville, Tenn. New York, D. Appleton & Co., 1, 3 & 5, Bond St., 1880.

This is an instructive and interesting work, imparting useful information in the improvement of soils, scientific agriculture, &c. It will well repay perusal.

INTERESTING EXERCISES IN QUALITATIVE ANALYSES: for Ordinary Schools, by Geo. W. Rains, M.D., Prof. Chemistry and Pharmacy in the Medical Department of the University of Georgia, and Instructor of Natural Science in the Richmond Academy, Augusta. New York, D. Appleton & Co., 1880.

"This work," the author remarks, "is designed to impart interest to the study of chemistry, &c., and will be found serviceable to medical students, druggists and physicians, enabling them, in many cases, by a speedy and simple process, to determine those salts and medicines which may have lost their labels. It is just the book for young amateurs."

THE POPULAR SCIENCE MONTHLY: Conducted by E. L. & W. J. Youmans. $5 per annum ; 50 cents per number. Volumes begin May and November each year. Subscriptions may begin at any time. D. Appleton & Co , New York.

Contains instructive and interesting articles and abstracts of articles, original, selected, and illustrated, from the pens of the leading scientific men of different countries; accounts of important scientific discoveries; the application of science to the practical arts ; the latest views put forth concerning natural phenomena, by savants of the highest authority.

PRACTITIONERS' REFERENCE BOOK: by Richard J. Dunglison, A. M. M. D., Editor Dunglison's Medical Dictionary, Secretary of the American Academy of Medicine, etc. Second edition, revised and enlarged. Philadelphia, Lindsay & Blakiston, 1880.

The edition of this very useful work here presented is much enlarged and improved, containing 475 ovtavo pages. As a work of reference and ready information upon a thousand items which a doctor needs to know, it is exceedingly reliable, and should be in the library of every physician.

LUCIE CODEY: A Novel, by Henry Greville, Author of "Dosia," "Marrying Off a Daughter," Savili's Expectation," etc. Translated by Mary Neal Sherwood. Philadelphia, T. B. Peterson &. Bros. Price 50 cents.

The above has been kindly sent us by the publishers for review. We are glad to receive books of any kind, but a medical journal cannot usually devote space for a review to other than medical and scientific works. We will, however, give title page and, perhaps, brief notice to any work sent to our office. Peterson & Bros. are extensive dealers in books, including novels, poems and standard works of the best authors.

THE MANAGEMENT OF CHILDREN IN SICKNESS AND IN HEALTH: A Book for Mothers, by Annie M, Hale. M.D. Philadelphia, Pressley Blakiston, 1012 Walnut St., 1880.

A practical and useful little work, containing many useful hints to mothers, who would do well to purchase the book and use as a guide in the management of babies, instead of following the thousand foolish suggestions of quacks and superstitious old women.

TRANSACTIONS OF THE AMERICAN GYNECOLOGICAL SOCIETY: Vol. 4 for the year 1879. Boston, Hougton, Mifflen & Co., 1880.

A large octavo volume of 500 pages, containing a number of able and interesting papers read before the Society at its fourth annual session at Baltimore, Sept 17, 1879.

